# Application of the suture anchor in the treatment of Hoffa fractures of the lateral femoral condyle

**DOI:** 10.1186/s13018-023-04005-y

**Published:** 2023-07-19

**Authors:** Yingya Jiao, Yanhui Suo, Junlin Chen, Ruihai Yan, Zhongqiang Yuan, Yinhu Shi, Cheng Chang, Meng Wei

**Affiliations:** 1 Department of Sports Injury and Arthroscopy, Handan City Central Hospital, Hebei, 056001 Handan China; 2 Department of CT Room, Handan City Central Hospital, Hebei Handan, 056001 China

**Keywords:** Suture anchor, External fixation, Femoral condyle, Hoffa fracture

## Abstract

**Background:**

To evaluate the feasibility and clinical effect of the suture anchor combined with external fixation in the treatment of the lateral femoral condyle Hoffa fracture.

**Methods:**

In this study, a retrospective study was conducted to analyze the feasibility of treating fourteen patients (eight men and six women) with Hoffa fractures admitted to our Hospital from January 2016 to October 2021 with combined external fixation using incisional reduction anchor nailing. The age of the patients ranged from 23 to 45 years, with an average of 37.5 years. According to Letenneur’s classification, there were eight cases of type I, three cases of type II, and three cases of type III. The functional assessment of Letenneur was used to measure the clinical outcome.

**Results:**

All patients had one-stage wound healing, and all patients were followed up for 12 to 18 months after surgery, and all fractures healed well, with normal knee flexion and extension activities, and no complications such as fracture displacement, anchor nail loosening, or fracture malunion were observed. The clinical outcome was evaluated according to the functional evaluation criteria of Letenneur et al. The clinical outcome of fourteen patients: excellent in thirteen cases and good in one case, with an overall excellent rate of 100%.

**Conclusions:**

Our study results indicate that the use of anchor nailing combined with external fixation for Hoffa fractures of the femoral condyle has some clinical reference significance because it is less invasive, has fewer complications, does not require secondary removal, and is worthy of clinical application.

*Trial registration*: Retrospectively registered.

## Background

Hoffa fracture refers to the fracture that occurs on the coronal plane of the femoral condyle, which was first described by Professor Friedrich Busch [1]. In 1904, it was officially named by Professor Albert Hoffa and then began to be systematically studied and classified [2]. The incidence of Hoffa fracture is low, accounting for 8.7% to 13% of distal femoral fractures [3]. Hoffa fracture is mainly caused by high-energy injury and more common in young adults, often involving the lateral femoral condyle. Hoffa fracture is an unstable intra-articular fracture, and is generally treated by surgery [4]. The anatomical reduction of the fracture and the integrity of the surrounding ligaments are important static factors that determine the stability of the knee joint recovery. Hoffa fracture often involves the attachment of the surrounding ligaments, so precise reduction of the fracture and preservation of ligament integrity in that area is crucial for the restoration of knee joint stability [5]. In the surgery, precise anatomical reduction and stable fixation are essential to avoid postoperative complications, such as deformity healing, traumatic arthritis and joint stiffness. In clinical practice, screws, plates or the combination of them are often used for the internal fixation [6]. However, when the dislocation of the articular surface is obvious, it is difficult to control the shear stress on the coronal plane simply by using screws [7]. In addition, when the articular surface collapses severely, the area where steel plates and lag screws can be placed is limited, making it difficult for operation, and a second operation is required to remove the internal fixation [8].

To overcome the above disadvantages in traditional internal fixation, we improved the surgical treatment for lateral femoral condyle Hoffa fracture by using the suture anchor combined with external fixation. In this study, we reviewed fourteen cases of Hoffa fractures of the femoral epicondyle treated with anchor nailing combined with external fixation (including tibial tuberosity traction or plaster) from January 2016 to October 2021 and further explored the feasibility of this procedure based on Letenneur evaluation criteria and postoperative follow-up results [9]. To understand the application and clinical significance of anchoring technique in Hoffa fracture of femoral condyle from a minimally invasive point of view.

## Methods

### Patient enrollment

Only descriptive statistics were used for the data of this study, and no comparison between groups was involved. We retrospectively analyzed fourteen cases of Hoffa fractures of the lateral femoral condyle (eight men and six women) admitted to the Handan Central Hospital from January 2016 to October 2021 and treated with anchor nailing combined with external fixation. The age of the patients ranged from 23 to 45 years, with an average of 37.5 years old. Among all patients, there were five cases with fracture on the left side and nine cases on the right side; three cases were injured by falling from height, and eleven cases were injured by car accident. All fractures were freshly closed fractures of the lateral femoral condyle, and there was no combined damage to other important organs, blood vessels and nerves. According to Letenneur’s classification [7], there were eight cases of type I, three cases of type II and three cases of type III. Open reduction was performed when the swelling of the injured area subsided, usually at 5 to 10 days after injury. In order to evaluate the displacement of the fracture, and determine whether there were some combined injuries such as ligaments and meniscuses damages, X-ray, spiral CT and MRI examination were routinely performed before surgery.

### Surgical technique

General anesthesia was administered and the patient was maintained at supine position. Initially, the fracture site, cruciate ligaments, meniscuses and other injuries were explored under arthroscopy. Two patients had lateral meniscus injury, and they were undergone partial resection of the meniscus under arthroscopy. All patients had no combined injury to the cruciate ligament and tibial plateau.

**Fig. 1:**
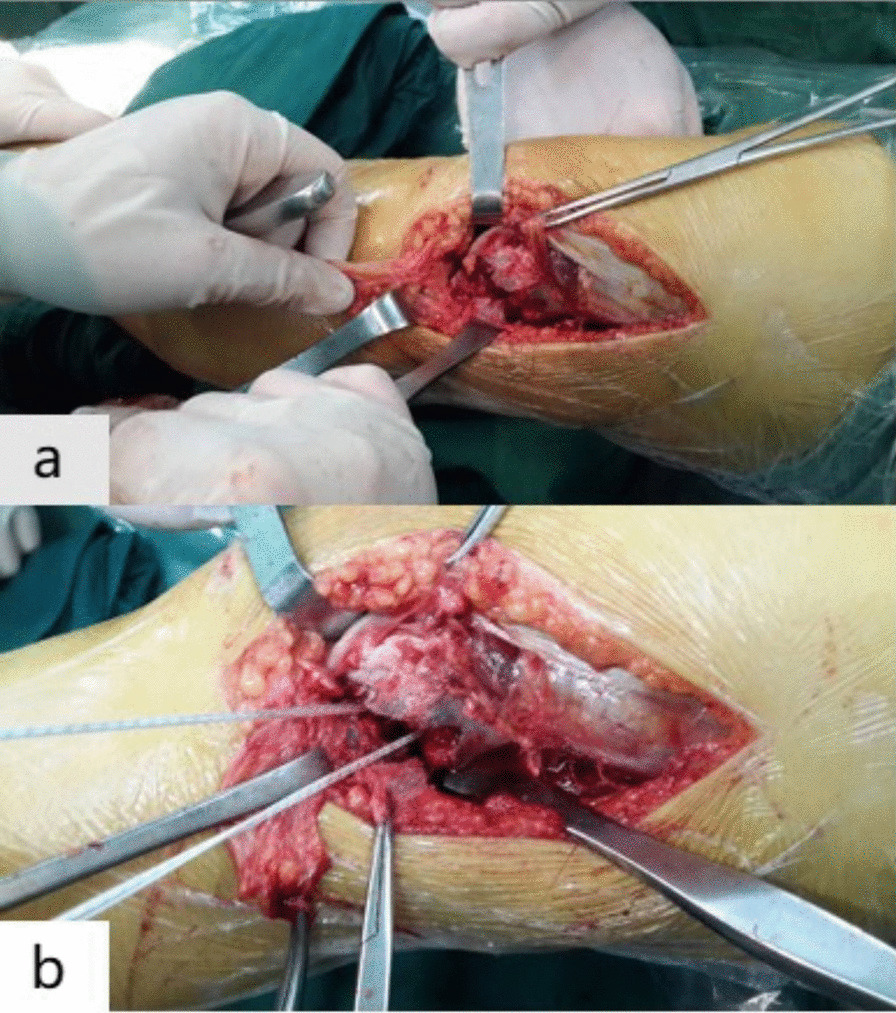
Exposure of the comminuted fracture of the lateral femoral condyle (**a**) and two suture anchors were screwed in the proximal end of the fracture (**b**)

**Fig. 2:**
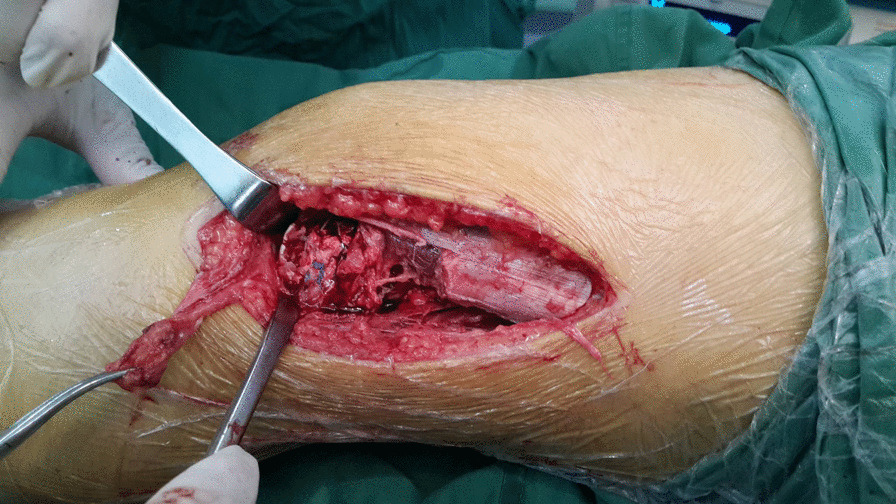
The anchor suture passed through the bone fragments and out of the cortical bone

After removing the arthroscopy, the knee was flexed 30°, and a posterolateral approach of the knee joint was adopted. With the lateral condyle as the center, the incision was extended longitudinally toward the proximal end for about 6 cm and then extended anterior and inferior toward the distal end for about 4 cm to the anterior upper edge of Gerdy’s tubercle. The iliotibial band was cut obliquely, and we went through the interval space between the lateral femoral muscle and the biceps femoris muscle to make the knee joint extreme varus, which could fully expose the number and size of fracture fragments and the collapse of the articular surface (Fig. 1a). The relatively complete section of the proximal bone bed was divided into three equal parts, and the two central positions were selected to screw in two suture anchors (3.5 mm or 5.0 mm, Smith & Nephew, USA). The anchor was placed in the direction of the harder cancellous bone (Fig. 1b). The anchor was screwed until the tail end was about 2 mm below the bone surface to avoid being pulled out, and the anchor should not penetrate the intercondylar fossa or penetrate into the joint cavity. The tail lines of the anchor were dispersed in multiple directions and in a parachute shape, and then, they were passed through the surrounding large bone fragments and out of the cortical bone. In this study, there were three patients, whose articular surfaces of the lateral condyle were severely collapsed. After reduction by leverage, autogenous iliac bone grafting was performed on the collapsed part to restore the curved contour of the articular surface. Then, eight anchor tail lines out of the cortical bone were tightened and knotted in pairs (Fig. 2). The deep fascia tissues such as ligaments around the cortical bone could be further sutured and strengthened with anchor tail lines.

**Fig. 3:**
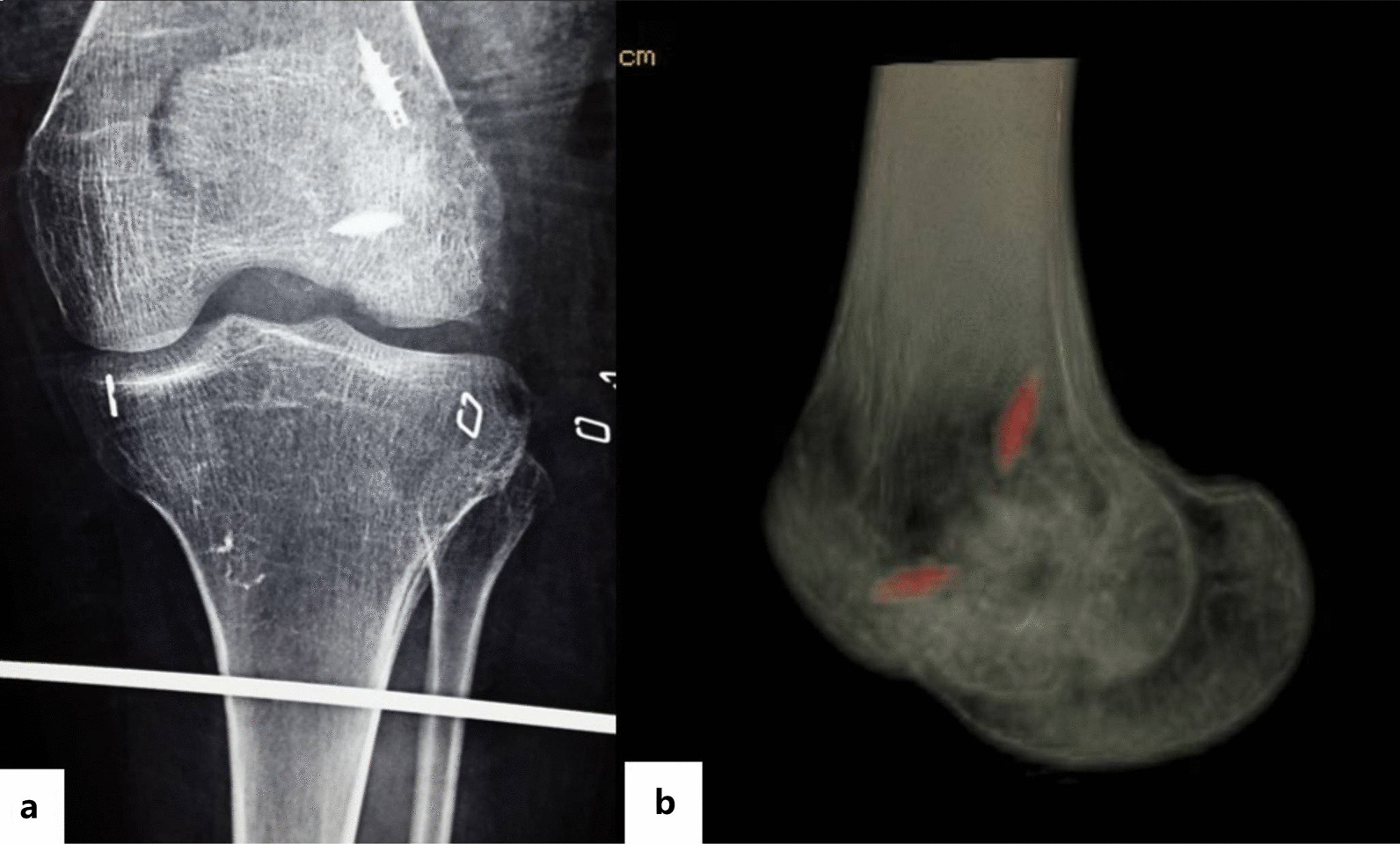
Postoperative tibial tuberosity traction was performed (**a**), and CT showed good positional alignment of fracture (**b**)

The fourteen patients with Hoffa fractures were all internally fixed with two suture anchors. By bone fragments reduction and bone grafting, the contour of the articular surface was effectively restored, and the severely collapsed intra-articular fractures were transformed into non-displaced comminuted fractures. However, the suture anchor belongs to the elastic internal fixation, which needs to be combined with tibial tuberosity traction (one case) (Fig. 3) or plaster external fixation (thirteen cases) to avoid re-compression of the collapsed site.

This research project was approved by the Ethics Committee of Handan Central Hospital, informed consent was obtained from the participants, and the principle of privacy protection was observed.

### Postoperative management

Routine prophylactic antibiotic broad-spectrum cefazolin sodium was used to prevent incision infection within 48 h after surgery. After waking up from anesthesia, the patient began to perform the exercises of ankle pump and isometric contraction of the quadriceps. The bone traction or plaster was removed and replaced with a dial-type activity limiting brace for protection at 4–5 weeks after the operation. Under the guidance of professional rehabilitation specialists, the knee joint extension and flexion activities were started step by step. The patient was partially weight bearing at 8 weeks after the operation. X-ray and CT were re-examined at 12 weeks after the operation, and full weight bearing began according to the fracture healing.

### Follow-up and evaluation of clinical outcomes

Patients were followed up for 12–18 months. The following Letenneur’s functional assessment was applied to measure the clinical efficacy: (1) excellent: knee joint flexion and extension range of motion > 120°, stable fracture, no pain in knee movement, no need to assist walking; (2) good: knee joint flexion and extension range of motion > 120°, stable fracture, mild pain in knee joint motion, but no affecting activities, can walk independently; (3) fair: the knee joint flexion and extension range of 90° ~ 120°, the fracture may be unstable, there is obvious pain after the activity, and need to assist walking; and (4) poor: knee joint flexion and extension range of motion < 90°, the fracture is unstable, often painful, and assistive devices are needed for walking.

## Results

In this study, there were fourteen patients, eight males and six females; age ranged from 23 to 45 years, mean age was 37.5 years; according to Letenneur’s classification: eight cases of type I, three cases of type II, and three cases of type III. All patients had rapid postoperative recovery, bony healing, fracture union, normal knee flexion and extension, and no postoperative complications such as fracture displacement, anchor nail failure, traumatic arthritis and joint stiffness. Among them, thirteen cases were excellent and one case was good, with an excellent rate of 100%, and the clinical efficacy was satisfactory.

### Case presentation

**Fig. 4:**
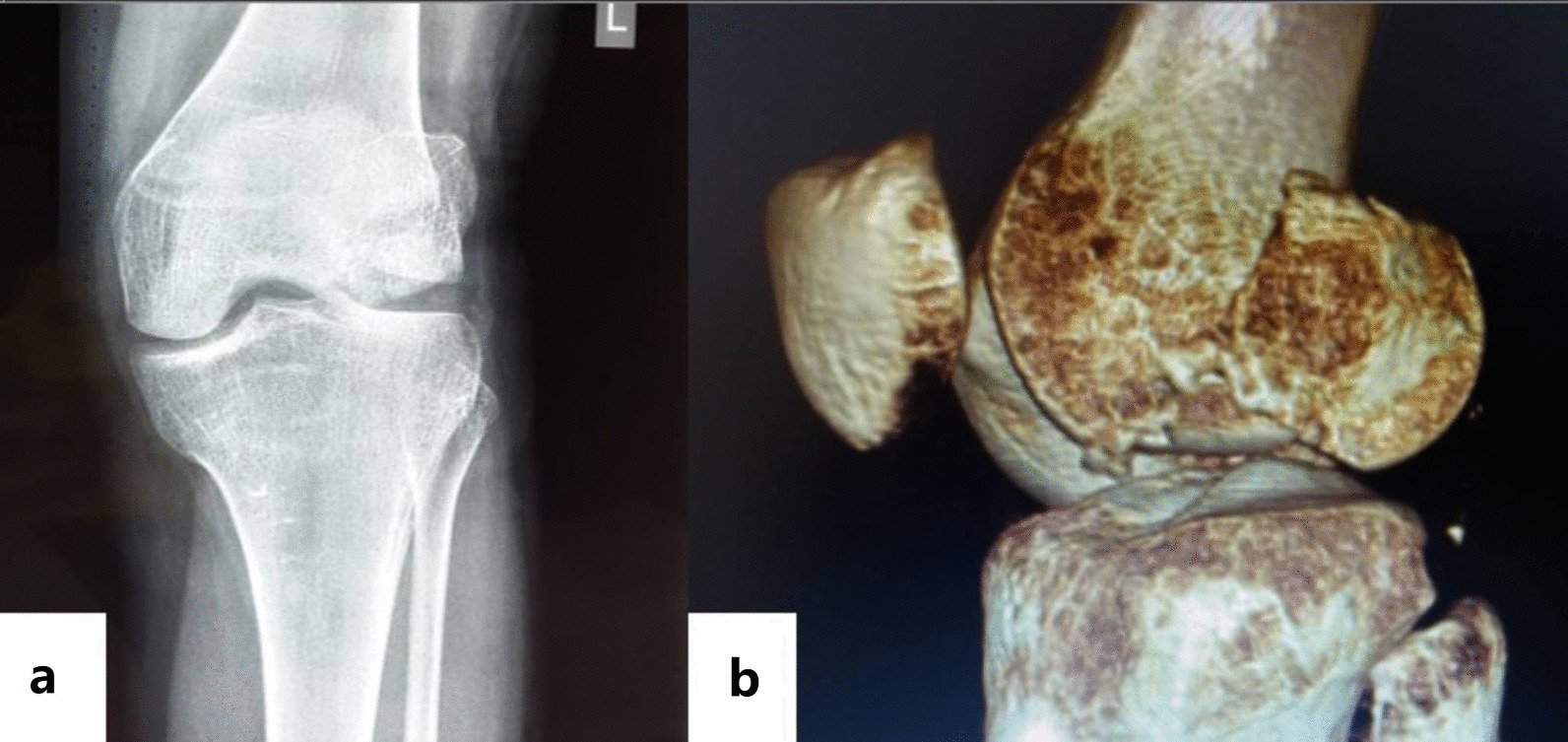
Preoperative X-ray (**a**) and CT showed severe fracture collapse (**b)**

**Fig. 5:**
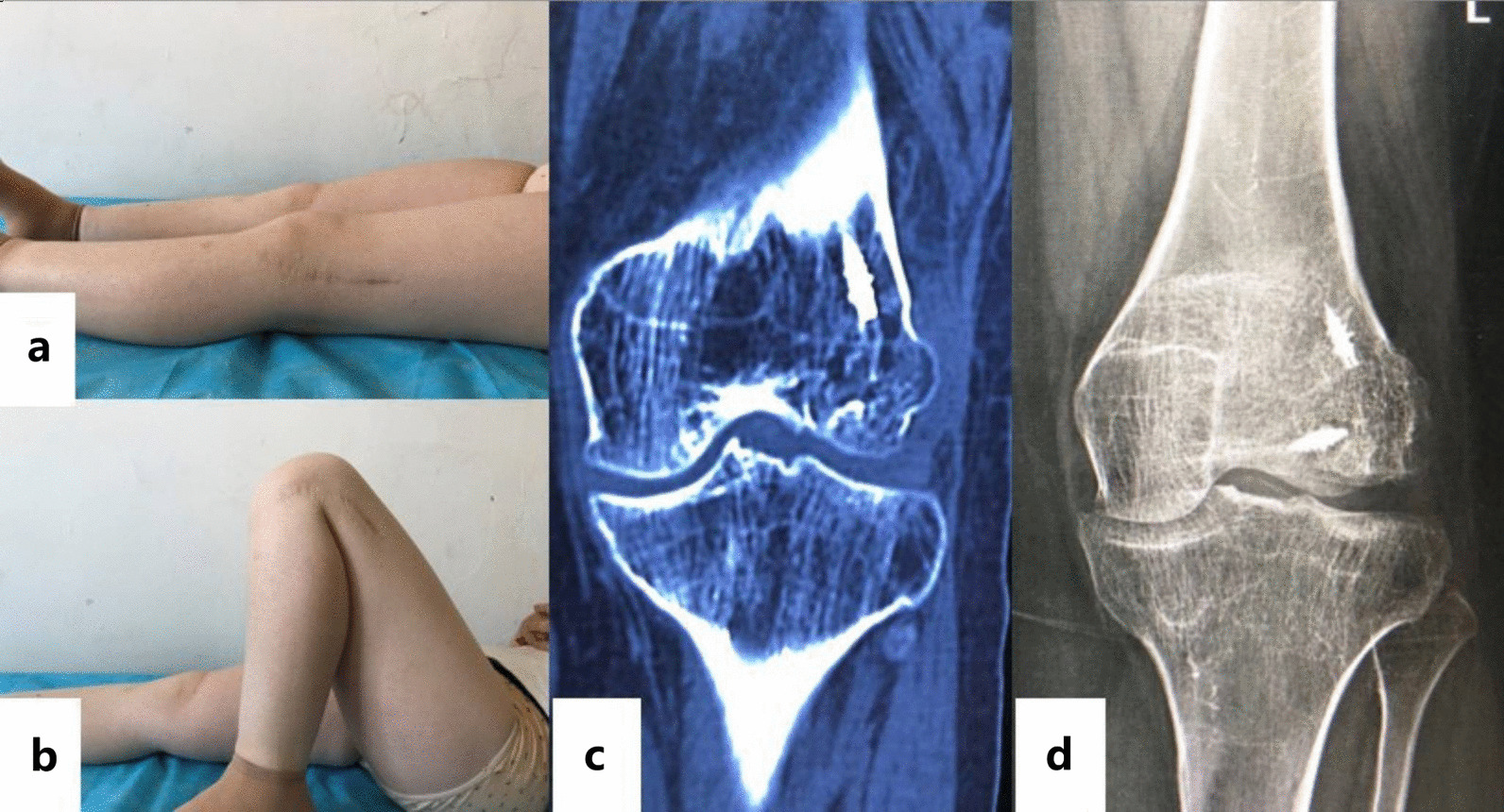
Knee range of motion at 18 months after surgery (**a**), (**b**) and CT (**c**), X-ray (**d**)

A 23-year-old female patient was admitted to the hospital in January 2016 due to a fall and was diagnosed with Hoffa fracture type I of the left lateral femoral condyle (Fig. 4a, b). A posterolateral approach of the left knee joint was adopted. Comminuted fracture of lateral condyle was seen, and the articular surface was severely collapsed. Two suture anchors were screwed in the proximal bone bed (Fig. 1b), with tail lines passed through the surrounding bone fragments. After reduction by leverage, autogenous iliac bone grafting was performed on the collapsed part. Tail lines were tightened and knotted after the joints were in good position (Fig. 2). Tibial tuberosity traction was used for 4 weeks and then replaced with a dial-type brace for protection (Fig. 3). The brace was removed, and the patient was partially weight bearing at 8 weeks after the operation. CT were re-examined at 12 weeks after the operation. CT indicated that the patient achieved fracture union, and full weight bearing began. X-ray, CT and range of motion of knee joint were re-examined at 18 months after the operation (Fig. 5a, b). Follow-up results showed that the range of motion of knee joint is restored and the Letenneur’s functional assessment of the patient was excellent (Fig. 5c, d).

## Discussion

Hoffa fractures have a low incidence in limb fractures and are unstable intra-articular fractures [10, 11]. The fracture line of the coronal plane in Hoffa fracture is consistent with the long axis of the lower extremity, and the shear stress acting on the posterior condyle is the direct cause of the displacement of Hoffa fracture [12]. There is a developmental valgus angle in the femoral shaft, which makes the lateral condyle more likely to fracture [13]. Therefore, the incidence of lateral femoral condyle fractures is much higher than that of the medial condyle. The articular bursa, ligament, muscle and tendon around the femoral condyle are attached, and severe fractures may even be accompanied by injuries of meniscus, articular cartilage, anterior cruciate ligament, posterior cruciate ligament, blood vessels and nerves. It is very important to protect the tissue around the knee joint during the operation. Local fixation of the fracture block with wire anchor with minimal trauma can reduce the peeling of the soft tissue around the fracture block. The lateral collateral ligament of the knee joint can even be strengthened and fixed by the tail line of the anchor, so the anatomical reduction of the fracture and the repair of the soft tissue of the knee joint is the key to the success of the operation [14].Surgical internal fixation is the best treatment for Hoffa fractures, and this treatment method can achieve anatomical reduction and stable internal fixation for early functional exercise [8].

Hoffa fractures are divided into three types based on the location and course of the fracture line according to the commonly used Letenneur’s classification [7]. Lewis et al. [15] further supplemented this classification based on the soft tissue attachment of posterior condyle fractures. Type I: A vertical fracture involving the entire posterior condyle and parallel to the posterior cortex of the femur. The fracture line passes through the cruciate ligament or the collateral ligament attachment point. Type II: Fracture parallel to the base of the condyle, the fracture line is behind the attachment point of the collateral ligament, and all or part of the gastrocnemius tendon or popliteal tendon is attached to the fracture line. Type III: Oblique fracture of the posterior condyle of the femur, with the fracture line anterior to the attachment point of the cruciate ligament. Li et al. [16] also proposed the CT classification of Hoffa fractures in 2013, which clarified the fracture line and fracture comminution. A perfect classification has important guiding significance for the diagnosis and treatment of Hoffa comminuted fractures.

In clinical treatment of Hoffa fractures, the commonly used internal fixation includes lag screws, Herbert screw and steel plates [17–19]. Jarit et al. [20] used screws to fix the posterior condyle. The head end of the screw should avoid piercing the contralateral articular cartilage, and the tail end needs to be countersunk or using Herbert screw to reduce the irritation to the cartilage surface. Lag screws can provide good compression between bone pieces, but they cannot effectively resist the shear stress of the coronal plane [18]. For the fracture with comminuted and collapsed part, the screw fixation alone is not very reliable, which will make the fracture easy to displace again and cause postoperative complications such as traumatic arthritis. From a mechanical point of view, placing an anti-sliding plate behind the femoral condyle is the best way to control the coronal shear stress [21]. Particularly for oblique fractures of the posterior femoral condyle of type III, hollow screws combined with supporting steel plates are often used clinically for internal fixation [22]. However, when placing the steel plate from the posterior articular surface, it is necessary to extensively strip the surrounding joint capsules and ligaments and other soft tissues, which is difficult to operate and severely damages the blood supply of the bone mass, easily leading to the occurrence of bone nonunion after surgery [23]. Moreover, the placement of the internal fixation is particularly difficult when the articular cartilage is severely collapsed, and a second operation is required to remove the internal fixation. For different types of Hoffa fractures, there is still a lack of consensus on how to choose the best implantation method of internal fixation [24].

The application of suture anchors for the treatment of Hoffa fractures has not been reported. In recent years, we have applied suture anchors to the treatment of Hoffa fractures and achieved good clinical results. The advantages of this treatment method are: (1) Regardless of Hoffa fracture type I, type II or type III, the posterolateral approach of the knee joint can be adopted. The operation is simple, and there is no need to strip too much surrounding tissue. (2) Two suture anchors are screwed into the cancellous bone at the proximal end, without damaging blood vessels and cartilage tissue, and there is no need to remove them with a second operation. (3) The multiple tail lines of the suture anchor pass through the surrounding bone fragments, which can accurately reduce the broken ends of the fracture. The severely collapsed area requires iliac bone grafting to restore the normal arc of the articular surface. In this study, there were 3 out of 8 patients received bone grafting because of the severely collapsed joint surface. This treatment method can ingeniously transform the collapsed and displaced fractures into undisplaced comminuted fractures. (4) The use of external fixation such as tibial tuberosity traction postoperatively can prevent the fracture from collapsing again. (5) When the bone fragment of the posterior condyle is large, one or two Kirschner wires can be cross-fixed in the lower segment of the femur from front to back. The wire tail is left outside the skin to facilitate outpatient removal. The above internal and external fixation methods effectively avoid the occurrence of postoperative complications such as traumatic arthritis, poor alignment of the lower limbs and joint adhesions. In this treatment method, although external fixation is needed for a period of time after the operation, the trauma during the operation is small and the impact on the joints is slight. Additionally, standard rehabilitation is applied after the removal of external fixation, so that the flexion and extension of the knee joint of the patient can be quickly restored.

The conventional Letenneur’s classification is divided into three types based on the anteroposterior positional relationship between the fracture line and the cruciate ligament, and considering the influence of blood supply [18, 25]. When encountering high-energy violent trauma, there is often a large area of collapse around the fracture line, which can involve multiple areas from type I to type III [26]. On the basis of the original classification, if the Hoffa fracture with a large-scale severe comminuted articular surface can be further defined as type IV, it will have important clinical significance for preoperative evaluation, intraoperative selection of internal fixation and prognosis.

## Conclusions

In conclusion, the application of wire anchor nailing combined with external fixation for Hoffa fractures of the femoral condyle restores the anatomical structure of the lateral condyle fracture surface, restores knee stability and avoids complications such as traumatic arthritis. The advantage of anchor nailing combined with the necessary external fixation is that the normal joint gap height can be maintained, while largely circumventing fracture fragment separation and traumatic arthritis caused by insufficient anchor nail fixation strength alone. Another advantage is that internal fixation with anchor nails avoids the need to remove the internal fixation twice, such as with conventional plates and common screws. This study has achieved satisfactory results, but the limitation is the low incidence of the disease and the limited number of surgical cases. The application of anchor nailing in the treatment of Hoffa fractures is rarely reported to the domestic and foreign literature, and further expansion of the sample size is needed in clinical practice. However, the present study also provides a treatment modality with less trauma, better repositioning and fewer complications from such patients, which has some clinical implications and reference value.

## Data Availability

All data generated or analyzed during this study are included in this published article.
